# Dataset of xerogel synthesis in basic medium at different resorcinol/catalyst ratios

**DOI:** 10.1016/j.dib.2018.02.041

**Published:** 2018-02-15

**Authors:** Rafael A. Fonseca-Correa, Liliana Giraldo, Juan Carlos Moreno-Piraján

**Affiliations:** a Facultad de Ciencias, Departamento de Química, Grupo de Investigación en Sólidos Porosos y Calorimetría, Universidad de los Andes, Bogotá, Colombia; b Facultad de Ciencias, Departamento de Química, Universidad Nacional de Colombia, Bogotá, Colombia

## Abstract

Textural properties of carbon xerogels and their applications depend on the path of synthesis. The variables to be considered are the starting reagents, relation and the catalysis medium used. Here we present a dataset for a series of carbon xerogels obtained by the polymerization of resorcinol-formaldehyde (R/F) at different resorcinol/Na_2_CO_3_ (C) ratios (R/C). To examine the effect R/C ratio on the morphology and surface chemistry of the xerogels, N_2_ isotherms at 77K, XRD, FTIR were measure as well as of immersion calorimetry measurements were taken in water and benzene; point zero charge (pzc) were also determined. These results are reported here.

**Specifications table**

**Table:** 

Subject area	Chemistry
More specific subject area	Materials Science: Carbon xerogels
Type of data	Tables, text, graphs.
How data was acquired	Isotherms of N_2_ 77 K (Quantachrome Inc. Sortometers IQ2), FTIR (Nicolet 6700, Thermo Scientific), XRD (Thermo Scientific), immersion calorimeter (“home-made”), pzc (Titrator SCHOTT TA20 plus)
Data format	Raw and analysed.
Experimental factors	Synthesis carbon xerogels in basic medium is performed using resorcinol/catalyst (R/C) at different ratios to determine these effects on textural, surface morphology and chemical properties of the materials obtained.
Experimental features	Four samples of carbon xerogels are synthesized at different R / C ratios [1], [2], [3]. These xerogels were characterized by adsorption-desorption isotherms of N_2_ at -77 K. Their textural properties were analyzed, and their PSD was evaluated using the NLDFT and QSDFT models using the IQ2 software. It was found that the xerogel with the lowest BET area corresponds to the lowest R/C ratio with a value of 133 m^2^.g^-1^ and the largest BET area is the xerogel synthesized with the highest R/C ratio, whose value is 830 m^2^ .g^-1^. The xerogels were analyzed with immersion calorimetry in water and benzene to determine the hydrophobicity of the prepared xerogels.
Data source location	Facultad de Ciencias, Departamento de Química, Universidad de los Andes (Bogotá, Colombia).
Data accessibility	Data are provided in this article

**Value of the data**

• The R/C ratio in the synthesis of carbon xerogels allows the scientist to design the most suitable routes to obtain characteristics that are suitable for the applications to obtain.
• Obtain a set of data on the textural properties, as chemistry are useful to analyze the effect of the ratios of the starting reagents.
• An adequate analysis of the data with the models established in the literature, allows the researcher to interpret the type of synthesized xerogels.
• The data obtained by immersion calorimetry complement the superficial and chemical information of carbon xerogels.

## 1 Data

### 1.1 Experimental design, materials and methods

#### 1.1.1 Materials

All chemical used in this data article were R.A. purchased from Aldrich™: Resorcinol (reagent ≥ 99.9%), Formaldehyde (37% w/w aqueous solution methanol stabilized) and deionised water.

#### 1.1.2 Synthesis of the organic xerogels

For the xerogels synthesised were employed two methods widely reported in the literature [1], [3]. Resorcinol (0.1120 mol) was dissolved in deionized water (W) at a constant ratio (R/W (0.075)). Then Na_2_CO_3_ (C) was added at different ratios (R/C): 25, 50, 800 and 1500, which acts as the basic reaction catalyst to accelerate the formation of the enolate ion Resorcinol. The formaldehyde solution was added to the resorcinol and catalyst solution at a ratio (R/F = 0.5) and agitated at 300 rpm for 30 min. The solutions were then placed in well-closed glass tubes (10 cm long x 1 cm internal diameter) and allowed to form the polymer gel: 1 day at 25 °C, 2 days at 50 °C and 3 days at 80 °C. The resulting resorcinol-formaldehyde (R/F) gels were dried under vacuum at 20 mm of mercury at 80 °C for 5 h (until no change in vacuum of the system was observed), thereby obtaining the resorcinol-formaldehyde (R/F) xerogels. To obtain carbon xerogels, the carbonization is carried out in a horizontal Carbolite furnace in N_2_ atmosphere at a flow rate of 100 mL/min and at a heating rate of 5 ° C/min to 850 °C. The sample are labeled: Xe25, Xe50, Xe800, and Xe1500.

#### 1.1.3 Experimental design

**Fig. 1:**
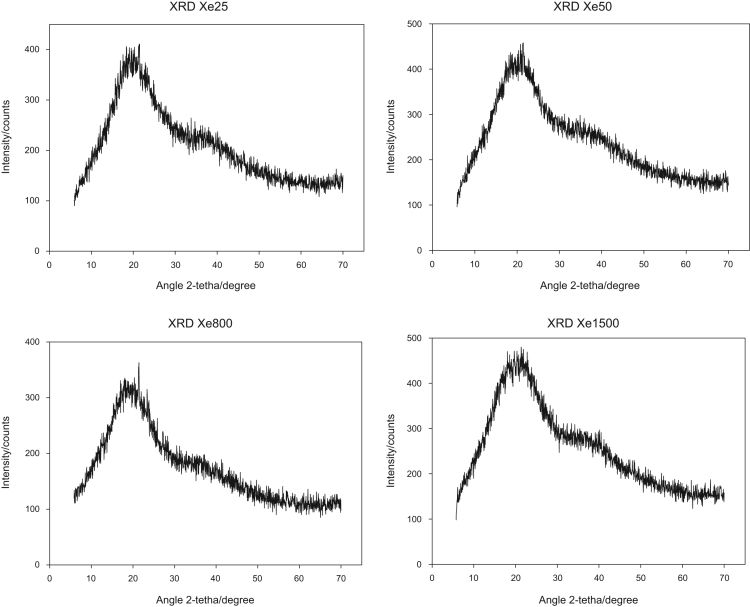
Diffractograms obtained by XRD for carbon xerogels.

**Fig. 2:**
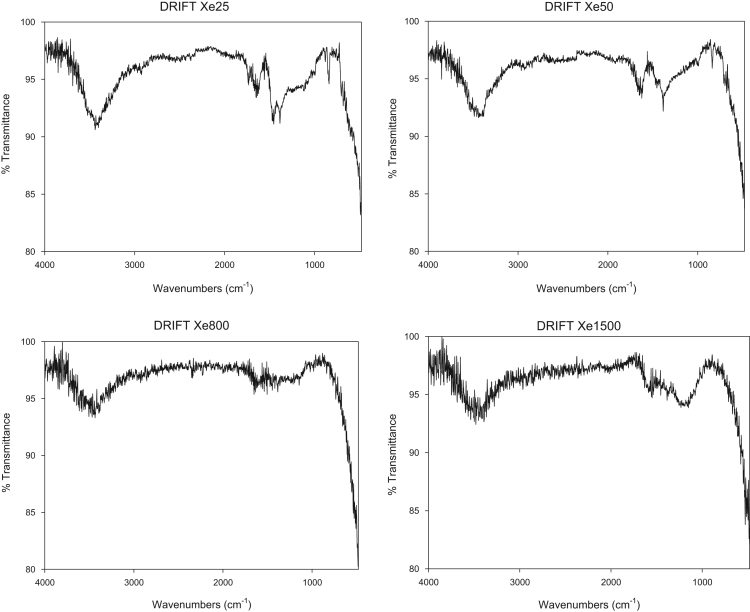
FTIR spectra for R/F carbon xerogels samples.

The dataset presented in Fig. 1, Fig. 2 respectively (XRD and FTIR) allow evaluate the structural and chemical differences during the change of R/C during the synthesis of carbon xerogels.

Table 1, Table 2, Table 3, Table 4 in the dataset obtained from adsorption isotherms of N_2_ at 77 K and some parameters are calculated with certain models applied widely in the literature. Dubinin-Raduskevich, Dubinin-Astakhov [4], BJH [5], BET models can be used [6], [7], and the Non-Local Density Functional Theory (NLDFT) and Quenched Solid Density Functional Theory (QSDFT) [8], [9] were applied to determine the Pore Size Distribution (PDS). In the Table 4 show of dataset of pH_pzc_ [10]. Table 1, Table 2, Table 3 show all the textural results of the synthesized samples, where the effect on the development of the microporosity and the mesoporosity and the R/C ratio is shown, as well as the values of pore volume that gives an indication of the porous structure of the samples. The adsorption energies are also observed for the N2 adsorption isotherms since it is an important parameter to determine the affinity of the adsorption of the samples.

**Table 1:** Textural parameters of carbon xerogels with hydrophobicity index.

**Carbon Xerogel**	**Method D-R**					**-∆H**_**imm**_**H**_**2**_**O/-∆H**_**imm**_**C**_**6**_**H**_**6**_
	Average width of pore [Å]	Energy of adsorption [kJ.mol^-1^]	Vmicropore [cm^3^.g^-1^]	S _micropore_ [m^2^.g^-1^]	Correlation Coefficient	HI^a^
**Xe25**	4.46	29.13	0.05	141	0.985	0.86
**Xe50**	6.98	18.62	0.06	173	0.998	0.93
**Xe800**	4.94	26.33	0.18	493	0.998	1.34
**Xe1500**	5.09	25.54	0.32	886	0.999	1.45

a HI=Hydrophobicity Index.

**Table 2:** Carbon xerogels: analysis of S_BET_, DA and BJH methods.

Carbon Xerogel	S_BET_ [m^2^ g^-1^]	D-A				B-J-H	
		V_micropore_ [cm^3^ g^-1^]	E_o_ [kJ mol^-1^]	n	Pore ratio [Å]	V_meso_ [cm^3^ g^-1^]	Pore ratio [Å]
**Xe25**	133	0.051	6.91	3.4	7.5	0.10	15.3
**Xe50**	173	0.086	4.85	1.3	8.0	0.16	17.1
**Xe800**	457	0.171	8.19	2.4	7.0	0.29	17.0
**Xe1500**	830	0.314	8.08	2.2	7.0	0.58	39.3

**Table 3:** Carbon xerogels: data report of PSD with kernels NLDFT and QSDFT, fitting error.

Carbon Xerogel	**NL-DFT**									
	Pore volume cyl [cm^3^ g^-1^]	Average width of pore cyl [Å]		E [%]	Pore volume cyl-slit [cm^3^ g^-1^]	Average width of pore cyl-slit [Å]	E [%]	Pore volume slit [cm^3^ g^-1^]	Average width of pore slit [Å]	E [%]
**Xe25**	0.14	5.84		1.80	0.14	3.52	1.51	0.14	4.30	1.48
**Xe50**	0.19	8.43		0.45	0.19	3.52	0.43	0.18	23.8	1.61
**Xe800**	0.35	6.64		2.84	0.35	3.52	2.76	0.35	3.93	0.98
**Xe1500**	0.78	5.25		0.44	0.77	3.12	0.42	0.74	3.59	1.14
	**QS-DFT**									
**Xe25**	0.14	5.33	1.15		0.14	3.47	0.96	0.14	3.07	1.34
**Xe50**	0.19	6.55	0.40		0.19	3.47	0.39	0.19	3.07	0.77
**Xe800**	0.36	5.33	0.59		0.36	3.47	0.52	0.35	3.07	0.67
**Xe1500**	0.76	5.15	0.74		0.75	3.20	0.72	0.75	3.07	1.05

**Table 4:** Carbon xerogels: point of zero charge (pzc).

SAMPLE	pHpzc
**Xe25**	6.88
**Xe50**	6.75
**Xe800**	6.45
**Xe1500**	6.40

The HI calculated from immersion calorimetry data are presented. [11], [12]. The hydrophobicity index correlates in a good way with the texture data.
